# 

**Published:** 1889-07

**Authors:** 


*Entered at the Post Office at Austin, Texas, as Second Class Mail Matter.*

Vol. V. JULY, 1889. No. 1.

### DANIEL'S TEXAS MEDICAL JOURNAL

Subscription, $2.00 a Year, in Advance. — Single Copies, Twenty-five Cents.

F. E. DANIEL, M. D., Editor and Publisher, AUSTIN, TEXAS.

